# Histone deacetylase inhibitor, butyrate, attenuates lipopolysaccharide-induced acute lung injury in mice

**DOI:** 10.1186/1465-9921-11-33

**Published:** 2010-03-20

**Authors:** Yun-Feng Ni, Jian Wang, Xiao-Long Yan, Feng Tian, Jin-Bo Zhao, Yun-Jie Wang, Tao Jiang

**Affiliations:** 1 Department of Thoracic Surgery, Tangdu Hospital, Fourth Military Medical University, Xi'an 710038, PR China

## Abstract

**Background:**

Histone deacetylase (HDAC) inhibitors, developed as promising anti-tumor drugs, exhibit their anti-inflammatory properties due to their effects on reduction of inflammatory cytokines.

**Objective:**

To investigate the protective effect of butyrate, a HDAC inhibitor, on lipopolysaccharide (LPS)-induced acute lung injury (ALI) in mice.

**Methods:**

ALI was induced in Balb/c mice by intratracheally instillation of LPS (1 mg/kg). Before 1 hour of LPS administration, the mice received butyrate (10 mg/kg) orally. The animals in each group were sacrificed at different time point after LPS administration. Pulmonary histological changes were evaluated by hematoxylin-eosin stain and lung wet/dry weight ratios were observed. Concentrations of interleukin (IL)-1β and tumor necrosis factor (TNF)-α in bronchoalveolar lavage fluid (BALF) and concentrations of nitric oxide (NO) and myeloperoxidase (MPO) activity in lung tissue homogenates were measured by enzyme-linked immunosorbent assay (ELISA). Expression of nuclear factor (NF)-κB p65 in cytoplasm and nucleus was determined by Western blot analysis respectively.

**Results:**

Pretreatment with butyrate led to significant attenuation of LPS induced evident lung histopathological changes, alveolar hemorrhage, and neutrophils infiltration with evidence of reduced MPO activity. The lung wet/dry weight ratios, as an index of lung edema, were reduced by butyrate administration. Butyrate also repressed the production of TNF-α, IL-1β and NO. Furthermore, the expression of NF-κB p65 in nucleus was markedly suppressed by butyrate pretreatment.

**Conclusions:**

Butyrate had a protective effect on LPS-induced ALI, which may be related to its effect on suppression of inflammatory cytokines production and NF-κB activation.

## Background

Acute lung injury (ALI) and acute respiratory distress syndrome (ARDS) are well defined and readily recognised clinical disorders caused by many clinical insults to the lung or because of predispositions to lung injury [1]. Sepsis and pneumonia are the main causes of ALI clinically. ALI occurring during gram-negative bacterial pneumonia and sepsis is caused in large part by lipopolysaccharide (LPS), a component of the cell walls of gram-negative bacteria [2]. When the cells in lung are exposed to LPS, the nuclear factor (NF)-κB is activated. NF-κB is a protein transcription factor that functions to enhance the transcription of a variety of genes, including cytokines and growth factors, adhesion molecules, immunoreceptors, and acute-phase proteins [3]. Upon activation by LPS, NF-κB is required for maximal transcription of many cytokines, including tumor necrosis factor (TNF)-α, interleukin (IL)-1β, IL-6, and IL-8, which are thought to be important in the generation of ALI. These cytokines and chemokines contribute to the vigorous recruitment of neutrophils in lung. Therefore, ALI is substantially caused by excessive neutrophil- and cytokine-mediated inflammation. Despite advancement in understanding the pathophysiology of ALI/ARDS and improved therapy methods, however, mortality rates of ALI/ARDS are around 40% [4].

Histone deacetylases (HDACs) regulate gene expression. In general, inhibitors of HDACs result in a nonspecific increase in gene expression. Therefore, they are considered as a new class of therapeutic agents for the treatment of tumor [5]. Agents such as trichostatin A (TSA) or suberoylanilide hydroxamic acid (SAHA) induce differentiation and/or apoptosis of transformed cells in vitro and inhibit tumor growth in vivo [6]. An unexpected effect of HDAC inhibitors, however, was revealed by recent studies indicating that they are able to suppress transcription and reduce inflammatory cytokines in models of autoimmune and inflammatory diseases [7,8]. Butyrate, a HDAC inhibitor, is a short-chain fatty acid derived from bacterial metabolism of dietary fibers in the colon and produces cell cycle arrest, differentiation and/or apoptosis of colorectal cancer cells in vitro [9-13]. Previous study has shown that butyrate reduced inflammation in Crohn's disease through NF-κB inhibition [14]. To date, unfortunately, the protective role of HDAC inhibitors in ALI is not well characterized.

The aims of this study were to investigate whether butyrate reduces inflammation in LPS-induced ALI in mice and to determine whether the protective effect is produced by suppression of inflammatory cytokines production and NF-κB activation.

## Materials and methods

### Animals and Reagents

Male BALB/C mice weighing 20-25 g were purchased from the Animal Center of the Fourth Military Medical University (Xian, China). All animals were allowed to take food and tap water ad libitum. All procedures were in accordance with the Declaration of Helsinki of the World Medical Association. The protocols were also approved by the Institutional Animal Care and Use Committee of the Fourth Military Medical University Tangdu Hospital. LPS (Escherichia coli lipopolysaccharide, 055:B5) and butyrate were obtained from Sigma Chemical Company (St. Louis, MO., USA), and were respectively dissolved in saline (1 mg/ml and 2 mg/ml). Enzyme-linked immunosorbent assay (ELISA) kits of TNF-α, IL-1β, myeloperoxidase (MPO) and nitric oxide (NO) were purchased from R&D Corporation (R&D Systems Inc. Minneapolis, MN, USA). Antibodies specific for total NF-κB p65, Lamin B and β-actin were obtained from the Wuhan Boster Biological Technology, Ltd. (Wuhan, China). Nuclear and cytoplasmic protein extraction kit was purchased from Beyotime Institute of Biotechnology (Beijing, China). BCA protein assay kit was obtained from Thermo Scientific Pierce Protein Research Products (Rockford, IL., USA).

### Experimental Protocol

All animals were randomly divided into 4 groups (n = 15, each group). Group 1 (control group) received an intratracheal injection of saline, group 2 (butyrate group) received an intragastric injection of butyrate (10 mg/kg), group 3 (LPS group) received an intratracheal instillation of LPS (1 mg/kg), and group 4 (LPS + butyrate group) received an intragastric injection of butyrate (10 mg/kg) 1 hour before LPS administration. At different time point (1, 3, 6, 12 and 24 hours) after administration, all animals were sacrificed. Bronchoalveolar lavage (BAL) was performed through the left lung. The superior lobe of right lung was excised for histopathologic examination. The middle lobe of right lung was excised for analysis of lung wet/dry weight ratio. The lower lobe of right lung was rapidly removed and cut into two parts in same size. A part of the lower lobe was homogenized and frozen in a cold phosphate solution at -80°C for MPO and NO analysis, and the another part was used to extract cytoplasmic and nuclear proteins for Western blot analysis.

### BAL

Animals were anesthetized with intraperitoneal pentobarbital (50 mg/kg). A median sternotomy allowed for exposure of both of the lungs. The trachea was exposed and inserted with an intravenous infusion needle. After ligating the hilum of right lung, the left lung was lavaged 5 times with 0.5 ml ice-cold phosphate buffered saline. The recovery ratio of the fluid was about 90%. The BAL fluid (BALF) was immediately centrifuged at 500 × g for 10 minutes at 4°C, and the cell-free supernatant was stored at -80°C for analysis of cytokines.

### MPO and NO Assays

To carry out the assays, tissue samples were subjected to three further freeze-thaw cycles and centrifuged at 12 000 × g for 10 minutes at 4°C. The supernatant was assayed for MPO activity and NO concentrations with ELISA kits. All procedures were done in accordance with the manufacturer's instructions.

### TNF-α and IL-1β Assays

Concentrations of TNF-α and IL-1β in BALF were measured by using ELISA kits. All procedures were done in accordance with the manufacturer's instructions.

### Lung wet/dry weight ratio

As an index of lung edema, the amount of extravascular lung water was calculated. The middle lobe of right lung was excised and the wet weight was recorded. The lung was then placed in an incubator at 80 °C for 24 hours to obtain the dry weight. And the wet/dry weight ratios were calculated by dividing the wet weight by the dry weight.

### Pulmonary Histopathology

The superior lobe of right lung was harvested at 24 hours after LPS administration and fixed with an intratracheal instillation of 1 ml buffered formalin (10%, PH 7.2). The lobe was further fixed in 10% neutral buffered formalin for 24 hours at 4°C. The tissues were embedded in paraffin and cut into 5 μm sections. Hematoxylin-eosin stains were performed using standard protocol.

### Western Blot Analysis

The lower lobe of right lung in each mouse was harvested separately at 1, 3, 6, 12 and 24 hours after LPS administration and frozen in liquid nitrogen immediately until homogenization. Tissue samples were homogenized and the cytoplasmic and nuclear proteins were extracted respectively according to instructions of Nuclear and Cytoplasmic Protein Extraction Kit. Protein concentrations were determined by BCA protein assay kit. Samples were separated on a denaturing 12% polyacrylamide gel and transferred to a nitrocellulose membrane. NF-κB p65 protein was detected by chemiluminescence using a rabbit polyclonal antibody according to the manufacturer's instructions.

### Statistical analyses

Data were entered into a database and analyzed using SPSS software, and expressed as means ± SEM. Statistically significant differences between groups were determined by ANOVA followed by Student's t test. Significance was accepted when p < 0.05.

## Results

### Effect of Butyrate on MPO Activity and NO Concentrations in Lung Tissues of Mice with ALI

**Figure 1:**
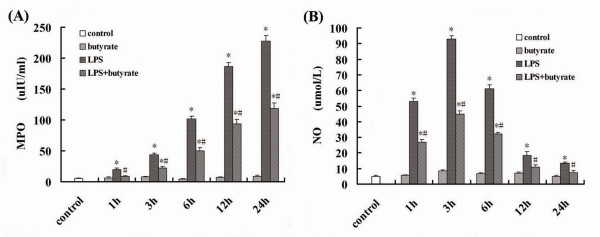
**Effect of butyrate on MPO activity and NO concentrations in lung tissues of mice with ALI**. A. The MPO activity in lung tissues at each time point after LPS administration and the effect of butyrate pretreatment. B. The concentrations of NO in lung tissues at each time point after LPS administration and the effect of butyrate pretreatment. Data are expressed as mean ± SEM, *P < 0.05 vs. control and butyrate group; ^#^*P *< 0.05 vs. LPS group.

After LPS administration, the MPO activity in lung tissues was significantly and continuously increased compared with the control and butyrate groups from 1 to 24 hours (fig. 1A). In addition, the concentrations of NO were significantly increased at 1 hour and peaked at 3 hours after LPS administration (fig. 1B). However, in LPS + butyrate group, butyrate pretreatment markedly decreased the MPO activity and NO concentrations at different time point (fig. 1A and 1B).

### Effect of Butyrate on the Concentrations of TNF-α and IL-1β in BALF of Mice with ALI

**Figure 2:**
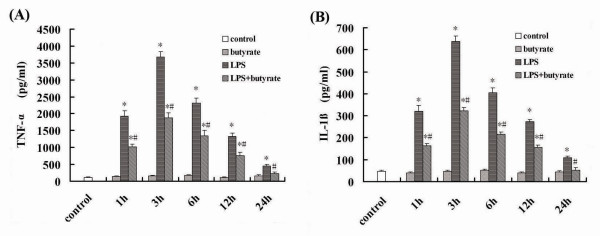
**Effect of butyrate on the concentrations of TNF-α and IL-1β in BALF of mice with ALI**. A. The concentrations of TNF-α in BALF at each time point after LPS administration and the effect of butyrate pretreatment. B. The concentrations of IL-1β in BALF at each time point after LPS administration and the effect of butyrate pretreatment. Data are expressed as mean ± SEM, *P < 0.05 vs. control and butyrate group; ^#^*P *< 0.05 vs. LPS group.

The concentrations of TNF-α and IL-1β in BALF were significantly increased at 1 hour and peaked at 3 hours after LPS administration (fig. 2A and 2B). Butyrate pretreatment efficiently reduced the production of TNF-α and IL-1β at different time point (fig. 2A and 2B).

### Effect of Butyrate on the Lung edema of Mice with ALI

**Figure 3:**
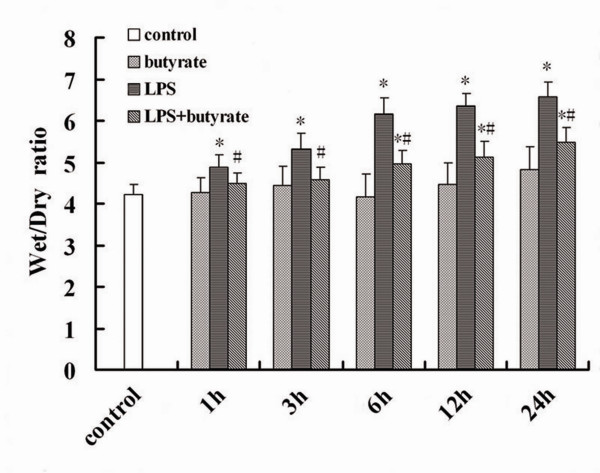
**Effect of butyrate on the lung edema of mice with ALI**. At each time point after LPS administration, pretreatment of butyrate decreased lung wet/dry ratios markedly. Data are expressed as mean ± SEM, *P < 0.05 vs. control and butyrate group; ^#^*P *< 0.05 vs. LPS group.

Compared with the control and butyrate groups, the lung wet/dry weight ratios were significantly and continuously increased from 1 to 24 hours after LPS administration. The increase of the lung wet/dry weight ratios was significantly reduced by butyrate administration at different time point (fig. 3).

### Effect of Butyrate on the Pulmonary Histopathological Changes of Mice with ALI

**Figure 4:**
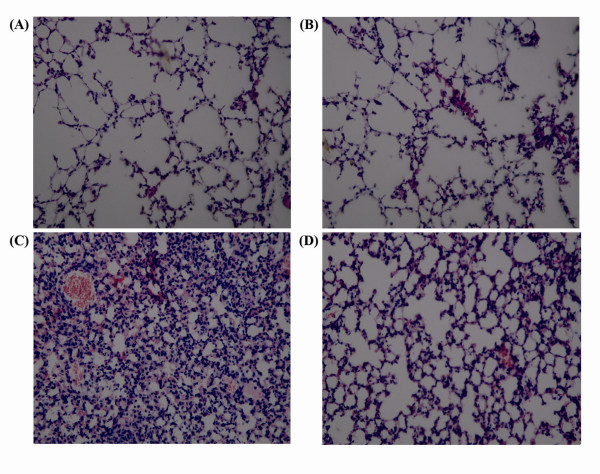
**Effect of butyrate on the pulmonary histopathological changes of mice with ALI**. Lung sections stained with hematoxylin-eosin from 24 hours after LPS administration revealed pulmonary histopathological changes (original magnification ×200). A. control group: normal structure. B. butyrate group: same as control group. C. LPS group: alveolar wall thickness, hemorrhage, alveolus collapse and obvious inflammatory cells infiltration. D. LPS + butyrate group: minor histopathological changes compared with LPS group.

Lung tissues from the control and butyrate groups showed a normal structure and no histopathological changes under a light microscope (fig. 4A and 4B). In LPS group, the lungs stained with hematoxylin-eosin indicated widespread alveolar wall thickness caused by edema, severe hemorrhage in the alveolus, alveolus collapse and obvious inflammatory cells infiltration (fig. 4C). In LPS + butyrate group, the histopathological changes of lung were minor compared with those in LPS group, especially in inflammatory cells infiltration (fig. 4D).

### Effect of Butyrate on the Activation of NF-κB in Lung Tissues of Mice with ALI

**Figure 5:**
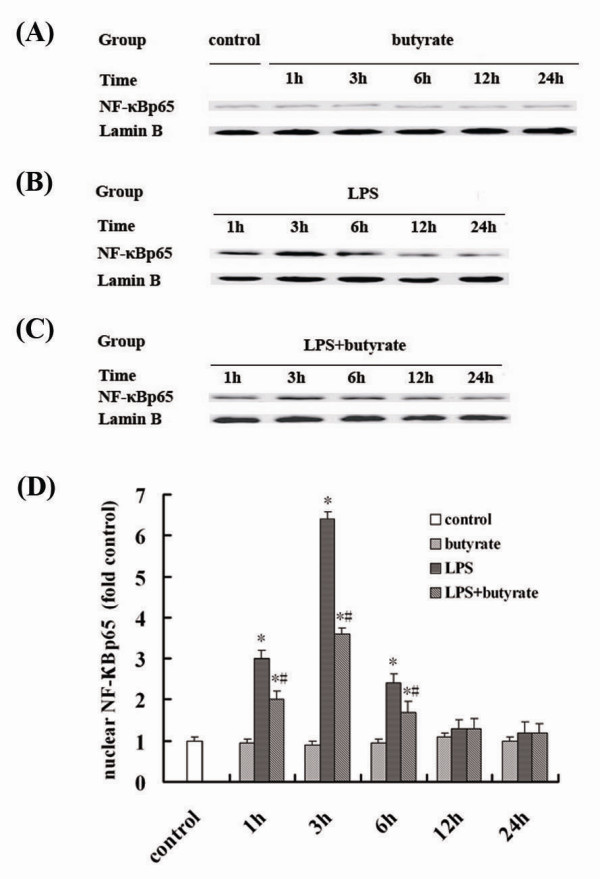
**Effect of butyrate on the nuclear expression of NF-κB in lung tissues of mice with ALI**. A., B. and C. Representative Western blots showed the nuclear expression of NF-κB p65 in lung tissues in different groups. D. Mean ± SEM NF-κB p65 optical densitometry from different groups. Pretreatment of butyrate significantly repressed the expression of NF-κB p65 in nucleus at 1, 3 and 6 hours after LPS administration. Data are expressed as mean ± SEM, *P < 0.05 vs. control and butyrate group; ^#^*P *< 0.05 vs. LPS group.

**Figure 6:**
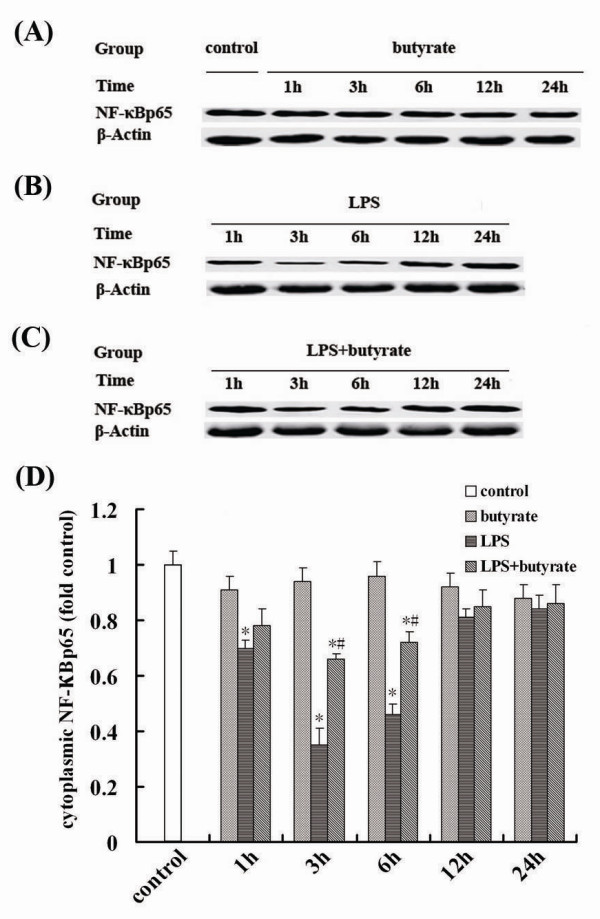
**Effect of butyrate on the cytoplasmic expression of NF-κB in lung tissues of mice with ALI**. A., B. and C. Representative Western blots showed the cytoplasmic expression of NF-κB p65 in lung tissues in different groups. D. Mean ± SEM NF-κB p65 optical densitometry from different groups. The expression of NF-κB p65 in cytoplasm was significantly reduced by LPS administration and these changes were inhibited by butyrate pretreatment. Data are expressed as mean ± SEM, *P < 0.05 vs. control and butyrate group; ^#^*P *< 0.05 vs. LPS group.

After LPS administration, the expression of NF-κB p65 in nucleus markedly increased and peaked at 3 hours (fig. 5B and 5D). The expression of NF-κB p65 in nucleus induced by LPS was significantly suppressed at 1, 3 and 6 hours by butyrate pretreatment (fig. 5C and 5D). On the contrary, the expression of NF-κB p65 in cytoplasm was significantly reduced by LPS administration (fig. 6B and 6D), and these changes were inhibited by butyrate pretreatment (fig. 6C and 6D). No changes of expression of NF-κB p65 in cytoplasm (fig. 6A and 6D) and nucleus (fig. 5A and 6D), same as control group, were observed with administration of butyrate alone at each time point.

## Discussion

The different results were observed in previous studies in vitro in terms of HDAC inhibitors' anti-inflammatory effects. ITF2357, a HDAC inhibitor, reduced IL-1, TNF-α and interferon-γ expression in LPS-stimulated human peripheral blood mononuclear cells [15]. Butyrate also reduced IL-12 production by human blood monocytes, and inhibited NO production in RAW macrophage cells [16,17]. However, pharmacological inhibition of HDAC increased the release of the pro-inflammatory cytokine IL-8 in human alveolar epithelial cells (A549) treated by TNF-α [18]. Moreover, TSA enhanced IL-8 production in SV-40-transformed lung epithelial cells stimulated by LPS [19].

Based on the results observed in lung epithelial cells, HDAC inhibitors seemed to be pro-inflammatory in ALI. In the present study, interestingly, our results indicated that administration of butyrate in vivo attenuated pulmonary inflammation in LPS-induced ALI. The reason for the discrepancy may be related to the different circumstances in vitro and in vivo. Furthermore, although the alveolar epithelial cells are the main part of lung tissues, the alveolar macrophages and neutrophils play a central role in ALI but not alveolar epithelial cells, which may contribute to the discrepancy. Consistent with the present study, TSA attenuated the development of allergic airway inflammation by decreasing expression of the Th2 cytokines, IL-4 and IL-5, reduced spinal cord inflammation, demyelination, neuronal and axonal loss, and ameliorated disability in the relapsing phase of experimental autoimmune encephalomyelitis, a model of multiple sclerosis [20,21]. Therefore, different from the variable results observed in vitro, HDAC inhibitors are more likely to exert their anti-inflammatory effects in vivo.

NF-κB pathway has been considered to play a pivotal role in the pathogenesis of ALI. NF-κB is normally retained in the cytoplasm in an inactive form through being associated with an inhibitor of κB (IκB) protein [22]. NF-κB is activated by a variety of pathogens known to cause inflammation and sepsis, including LPS. Following activation, the IκB protein breaks down and liberates NF-κB to enter the nucleus where it binds to specific sequences in the promoter/enhancer regions of genes [23,24]. Thus, NF-κB improves the transcription of most pro-inflammatory molecules, including adhesion molecules, enzymes, cytokines, and chemokines. Among these pro-inflammatory molecules, TNF-α and IL-1β are the most important cytokines in the pathogenesis of ALI. Elevated concentrations of TNF-α and IL-1β have been measured in BALF from patients with ARDS, and were related to prognosis [25,26]. Because TNF-α and IL-1β can stimulate the production of a variety chemotatactic cytokines such as IL-8, epithelial cell neutrophil activator (ENA-78), monocyte chemotactic peptide, and macrophage inflammatory peptide-1a (MIP-1a), they have earned a position of prominence at the head of the inflammatory cytokine cascade [27]. Therefore, the inhibition of TNF-α and IL-1β showed the reduction of pulmonary injury in ALI induced by LPS in mice [28,29].

In the present study, the concentrations of TNF-α and IL-1β in BALF and the expression of NF-κB p65 in nucleus increased significantly after LPS administration, and reached their peak at 3 hours respectively. Pretreatment of butyrate markedly reduced the concentrations of TNF-α and IL-1β in BALF, and suppressed the expression of NF-κB p65 in nucleus. In addition, we also found that in ALI mice the elevated MPO activity, a specific granulocyte enzyme, was significantly reduced by butyrate pretreatment. Although neutrophils have beneficial actions in eradicating microbial infections, excessive neutrophil accumulation in lung contributes to the development of ALI. The cytokines secreted by alveolar macrophages, such as TNF-α and IL-1β, play a key role in neutrophil recruitment to the lung. Therefore, the reduced neutrophils infiltration in lung by butyrate administration was partially due to its inhibitory effect on cytokines production.

In our study, we also found the reduced expression of NF-κB in cytoplasm due to the enhanced NF-κB nuclear translocation after LPS administration, which was markedly inhibited by butyrate pretreatment. Previous study showed that butyrate pretreatment of a human colon cell line (HT-29 cells) inhibited the TNF-α-induced nuclear translocation of the pro-inflammatory transcription factor NF-κB in part by preventing the complete degradation of IκB-α by reducing proteasome activity in the cell [30]. Moreover, butyrate decreased TNF production and pro-inflammatory cytokine mRNA expression by intestinal biopsies and lamina propria cells from Crohn's disease patients, and abolished LPS induced expression of cytokines by peripheral blood mononuclear cells and transmigration of NF-κB from the cytoplasm to the nucleus [14]. In the RAW 264.7 murine macrophage cells stimulated by LPS, butyrate down-regulated NO production and prevented the activation of NF-κB through the stabilization of IκB-α and IκB-β [31]. These results observed in vitro suggested that the anti-inflammatory effects of butyrate in ALI may primarily rely on inhibition of IκB degradation, and consequently inhibiting the nuclear translocation of NF-κB and the production of cytokines regulated by NF-κB.

Pulmonary edema is a life-threatening condition that frequently leads to acute respiratory failure. Injury to the alveolar epithelium can disrupt the integrity of the alveolar barrier or down-regulate ion transport pathways, thus, reducing net alveolar fluid reabsorption and enhancing the extent of alveolar edema [32]. Here we observed a significant reduction of pulmonary injury and edema in lungs of ALI mice treated by butyrate. Therefore, butyrate possessed the protective effect on ALI, which implied the clinical use of butyrate in future.

In the present study, the administration of butyrate was applied orally not intranasally. Butyrate is an anti-tumor agent, so it is cytotoxic. Therefore, the intranasal delivery of butyrate may be harmful to the nasal mucosa. Furthermore, the nasal absorption of butyrate by each mouse would be different due to the influence of respiration. Because of these, we considered that intragastric injection of butyrate might be a more appropriate form of administration in this study. Moreover, our results showed that this form of administration was feasible and effective. In addition, we only investigated the protective effect of butyrate on ALI on the condition that butyrate was administrated before LPS exposure, which was not often possible clinically. Given that pro-inflammatory cytokines TNF-α and IL-1β were released within hours after onset of ALI, early butyrate treatment after LPS exposure should be protective. Thus, in the further study, we will evaluate the protective effect of delayed butyrate treatment on ALI, and also investigate the optimal time window and dose of delayed butyrate treatment.

In conclusion, we have provided the first evidence that pretreatment of butyrate significantly attenuated pulmonary inflammation in LPS-induced ALI in mice. Thus, they might be useful in situations where current anti-inflammatory therapies are unsatisfactory.

## Abbreviations

ALI: acute lung injury; HDAC: histone deacetylase; LPS: lipopolysaccharide; IL: interleukin; TNF: tumor necrosis factor; BALF: bronchoalveolar lavage fluid; NO: nitric oxide; MPO: myeloperoxidase; ELISA: enzyme-linked immunosorbent assay; NF-κB: nuclear factor-κB; ARDS: acute respiratory distress syndrome; TSA: trichostatin A; SAHA: suberoylanilide hydroxamic acid; IκB: inhibitor of κB; ENA: epithelial cell neutrophil activator; MIP: macrophage inflammatory peptide.

## Competing interests

The authors declare that they have no competing interests.

## Authors' contributions

WYJ and JT designed the study and performed analyses. NYF performed the experiment, interpreted the data, and wrote the manuscript. WJ performed experimental measurements and helped to write and revise the manuscript. YXL performed experimental measurements and helped to write the manuscript. TF and ZJB helped with the animal experiment and experimental measurements. All authors read and approved the final manuscript.
